# The Relationship of Cognitive Performance and the Theta-Alpha Power Ratio Is Age-Dependent: An EEG Study of Short Term Memory and Reasoning during Task and Resting-State in Healthy Young and Old Adults

**DOI:** 10.3389/fnagi.2017.00364

**Published:** 2017-11-07

**Authors:** Janet P. Trammell, Priscilla G. MacRae, Greta Davis, Dylan Bergstedt, Ariana E. Anderson

**Affiliations:** ^1^Division of Social Sciences and Natural Sciences, Seaver College, Pepperdine University, Malibu, CA, United States; ^2^Department of Psychiatry and Biobehavioral Sciences, University of California, Los Angeles, Los Angeles, CA, United States; ^3^Department of Statistics, University of California, Los Angeles, Los Angeles, CA, United States

**Keywords:** cognition, aging neuroscience, Theta Alpha Ratio, EEG, aging

## Abstract

**Objective:** The Theta-Alpha ratio (TAR) is known to differ based upon age and cognitive ability, with pathological electroencephalography (EEG) patterns routinely found within neurodegenerative disorders of older adults. We hypothesized that cognitive ability would predict EEG metrics differently within healthy young and old adults, and that healthy old adults not showing age-expected EEG activity may be more likely to demonstrate cognitive deficits relative to old adults showing these expected changes.

**Methods:** In 216 EEG blocks collected in 16 young and 20 old adults during rest (eyes open, eyes closed) and cognitive tasks (short-term memory [STM]; matrix reasoning [RM; Raven's matrices]), models assessed the contributing roles of cognitive ability, age, and task in predicting the TAR. A general linear mixed-effects regression model was used to model this relationship, including interaction effects to test whether increased cognitive ability predicted TAR differently for young and old adults at rest and during cognitive tasks.

**Results:** The relationship between cognitive ability and the TAR across all blocks showed age-dependency, and cognitive performance at the CZ midline location predicted the TAR measure when accounting for the effect of age (*p* < 0.05, chi-square test of nested models). Age significantly interacted with STM performance in predicting the TAR (*p* < 0.05); increases in STM were associated with increased TAR in young adults, but not in old adults. RM showed similar interaction effects with aging and TAR (*p* < 0.10).

**Conclusion:** EEG correlates of cognitive ability are age-dependent. Adults who did not show age-related EEG changes were more likely to exhibit cognitive deficits than those who showed age-related changes. This suggests that healthy aging should produce moderate changes in Alpha and TAR measures, and the absence of such changes signals impaired cognitive functioning.

## Introduction

From 2014 to 2060, the number of older adults in the U.S. population is expected to more than double (US Census Bureau, 2016), an increase that will likely lead to parallel increases in the number of age-related chronic diseases. For instance, the prevalence of dementia—a disease with annual costs estimated to be $157–$215 billion (Hurd et al., 2013)—increases from 1 in 20 persons for those 71–79 years of age, to 1 in 3 persons for those over 90 years of age (Plassman et al., 2007). With aging, there is a linear decline in executive function beginning in the third decade of life, despite the fact that overall acquired knowledge (crystalized intelligence) continues to improve through the first five decades of life (Salthouse, 2012). Therefore, understanding cognitive function in old adults and being able to identify brain activity associated with optimal cognitive performance could lead to the development of better prevention and treatment of dementia and cognitive impairment.

Changes in cognition with aging have been examined in numerous studies, but the nature of this relationship has only more recently been examined with electroencephalography (EEG) technology. These studies have examined age-related changes in cognition and EEG metrics, often with inconsistent results. Cognitive functioning, as measured by memory, declines with increasing age (e.g., Gilbert and Levee, 1971; Salthouse, 1990; Parkin and Walter, 1991; Yokota et al., 2000; Hartman et al., 2001; Salat et al., 2002), but the EEG metrics associated with or underlying this decline are poorly understood. For instance, Delta (1–4 Hz) relative power has been found to correlate with cognitive performance with inconsistent results. Vlahou et al. (2014) found Delta power positively associated with executive function and perceptual speed in old, but not young adults, suggesting that associations between cognitive performance and Delta power may depend upon age. Finnigan and Robertson (2011) did not show a relationship between Delta relative power and any cognitive measure (recall, attention, executive function) in 73 healthy old adults (mean age 60) who had subjectively complained of memory loss but had no objective measures of memory dysfunction.

Other EEG metrics, such as Alpha (8–12 Hz), are also associated with cognitive performance. Individual Peak Alpha Frequency (iPAF), the peak of spectral Alpha power of the EEG, is generally positively correlated with memory and attention at all ages (Klimesch, 1997, 1999; Angelakis et al., 2004; Clark et al., 2004; Grandy et al., 2013). However, Finnigan and Robertson (2011) found no relationship between iPAF and cognitive performance in old adults. Independent of neurocognitive performance, Alpha rhythms decrease as a function of age (Chiang et al., 2011), with even more dramatic change in Alpha rhythms seen in neurodegenerative disorders leading to dementia such as Huntington's disease (Streletz et al., 1990) and Alzheimer's disease (Montez et al., 2009; Basar and Guntekin, 2013). This suggests that age should modulate the associations of cognitive ability with Alpha power.

Theta (4–8 Hz) power studies of cognitive ability have also generated less-consistent age-dependent findings. Significant positive correlations were reported between Theta power and cognitive deficits in healthy adults (Jelic et al., 1996) and higher baseline Theta power was found to be indicative of subsequent cognitive decline (Jelic et al., 2000; Prichep et al., 2006). In contrast, others reported Theta power positively associated with memory, executive functioning, perceptual speed, reasoning, and attention in old adults (Cummins and Finnigan, 2007; Cummins et al., 2008; Finnigan and Robertson, 2011; Vlahou et al., 2014). During the encoding phase of a spatial navigation task (cognitive mapping), Lithfous et al. (2015) found that Theta activity positively correlated with accuracy for young but not old adults. In comparison to young adults, old adults showed both reduced accuracy and reduced Alpha and Theta during encoding.

In addition to separate Theta and Alpha band analyses, their ratios have been more recently implicated as a potentially important indicator of cognitive ability in old adults. In older adults with amnesic mild cognitive impairment (aMCI), the Theta to Alpha Ratio (TAR) was increased relative to controls (Bian et al., 2014), findings echoed closely by (Moretti, 2015). The reverse metric, the Alpha to Theta Ratio, was used to discriminate individuals with probable Alzheimer's disease from healthy older controls (Schmidt et al., 2013), and differed within patients with mild and severe Alzheimer's disease (Penttilä et al., 1985). Furthermore, the Alpha 1 (8–10 Hz) to Theta Ratio was able to discriminate individuals with and without cognitive impairment in older individuals with Parkinson's disease (Bousleiman et al., 2015). Differences in the TAR metric between young and old adults has not yet been examined. In sum, changes in Theta and Alpha bands are likely predictive of cognitive impairment (Klimesch, 1999; Fonseca et al., 2011) in old adults, but this relationship may depend upon age.

To investigate the relationship between aging, EEG metrics (Theta, Alpha, and TAR) and cognitive performance (short term memory and reasoning), we modeled these relationships in 36 healthy adults, 16 young and 20 old, using EEG across six different blocks (Resting and Active states). Given the associations of cognitive performance with Theta/Alpha levels within old adults, we hypothesized that EEG signatures previously associated with cognitive functioning may differ within healthy young and old adults. The outcome measure evaluated was TAR in three midline regions (Fz, Cz, Pz). Furthermore, we assessed whether the correlations between EEG metrics (iPAF, relative Alpha, relative Delta, and relative Theta) and cognitive performance were the same *during* the cognitive tasks as at rest, as most research has investigated the relationship only between *resting* EEG metrics and cognition. Collectively, this manuscript identifies whether EEG signatures of increased cognitive performance are consistent within healthy old and young adults, and whether aging changes these signatures in the absence of any overt pathology.

## Method

### Participants

Sixteen young adults (20.7 ± 0.9 years, range 20–29 years of age, 8 women and 8 men) and 20 high functioning old adults (72.9 ± 2.5 years, range 70–79 years of age, 14 women and 6 men) completed the study after signing informed consent approved by the Institutional Review Board of Pepperdine University. Young adults were recruited from the university via on-campus advertisements. Old adults were recruited from the local community via advertisements in the local senior center newsletter. All participants received a $20 gift card to a local grocery store for participating in the study.

### Procedures

Young and old participants completed a questionnaire about their health history. Participants diagnosed with a concussion, stroke, epilepsy, neurological disease (dementia, Parkinson's disease, schizophrenia), or diabetes; or who experienced a heart attack, congestive heart failure, or cancer in the last year; or who were currently taking hypertensive or psychotropic medications, were excluded from the study. After passing the initial health history screening, qualified participants were asked to refrain from consuming alcohol, caffeinated beverages, and any other central nervous system stimulants for 4 h prior to the cognitive assessment session. Once the participant arrived at the lab, he/she gave informed consent and was screened for cognitive impairment, depression, and visual deficits. In order to be included in the study, participants were required to pass the cognitive assessment, scoring >26 (range 0–30) on the Mini Mental Status Examination (MMSE; Folstein, 1975); the depression assessment, scoring <4 on the abbreviated 15-item Geriatric Depression Scale (GDS; Sheikh and Yesavage, 1986); and demonstrate normal or corrected-to-normal vision (20/20). All 16 of the young adults successfully passed all screens and 20 out of 21 old adults passed. One older woman was excluded for scoring below 26 on the MMSE. Following these screenings, each participant prepared for EEG recording and completed the cognitive tasks (described below). The session took approximately 90 min.

### EEG recording

Each participant was seated in a dimly lit room and fitted with an electrode cap. EEG was sampled with 19 electrodes in standard 10–20 International Electrode System placements (FP1, FP2, F7, F3, Fz, F4, F8, T3, C3, Cz, C4, T4, T5/P7, P3, Pz, P4, T6/P8, O1, O2), with reference to linked ears. Impedance was maintained below 10 kOhms and within 1.5 kOhm difference between sites. EEG data was collected using a Mitsar 201 M amplifier, EEGStudio v1.6/WinEEG v.2.103.70 software (Mitsar Ltd., St. Petersburg, Russia), and electrode caps (Electro-Cap Intl. Inc., Eaton, OH).

EEG recording involved separate recording blocks in the following order: an initial 5-min eyes-open resting baseline (EO1, block 1), an initial 5-min eyes-closed resting baseline (EC1, block 2), randomly ordered cognitive tests (matrix and short term memory tests, blocks 3 and 4), a final 5-min eyes-open resting baseline (EO2, block 5), and a final 5-min eyes-closed resting baseline (EC2, block 6). EEG data was plotted, filtered (bandpass: 0.1–30.0 Hz, notch: 55.0–65.0 Hz), and carefully inspected using manual artifact-rejection for all tasks. Episodic artifacts including eye blinks, eye movements, jaw tension, body movements, and EKG interference were removed from all channels by two trained researchers and subsequently reviewed by a third researcher to reach consensus on any discrepancies. Relative power was computed by dividing the band specific values by total power. The data was divided into epochs of 500 samples of continuous artifact free data (2 s). The WinEEG spectral analysis tool was used to determine EEG relative power activity in the following frequency bands: Delta (0.5–4.0 Hz), Theta (4.0–8.0 Hz), Alpha (8.0–12.0 Hz), and Beta (12.0–24.0 Hz), during EC1 and during the cognitive tasks. iPAF was determined by evaluating the maximal difference peak (6.0–13.5 Hz) in occipital and parietal electrode sites during Alpha suppression (EC1-EO1). A natural log transform was applied to all EEG variables to normalize the data distribution.

Since Alpha rhythm changes with age, memory load, and pathology, the logarithm of the Theta/Alpha ratio was calculated within each block for each electrode using the maximum power within two frequency bands for Alpha: Alpha 1 (8–10 Hz.) and Alpha 2 (10–12 Hz.) as recommended by Klimesch (1999) and Haegens et al. (2014). This allows the Alpha power to vary based upon activity and age, yet constrains it within the frequency band of the individual peak Alpha frequency for all but two participants. These models were additionally replicated when removing the two older participants whose iPAF of 7.5 Hz were outside the 8–12 Hz region, to assess whether deviations in peak Alpha may unduly influence the relationship between cognitive ability and the TAR. Finally, all models were also replicated using Alpha which was set uniformly at 8–12 Hz, to assess whether a fixed Alpha bandpower would identify a similar relationship to a variable Alpha.

### Cognitive tasks

The cognitive assessments were completed on a computer using E-Prime® software. Participants completed two cognitive tasks: Short Term Memory (STM) and Raven's Matrices (RM). The cognitive tasks were counterbalanced for each participant and occurred during EEG blocks 3 and 4. In STM, participants were shown a list of 12 words (see Appendix A in Supplementary Material) in random order appearing sequentially for 1 s each. Immediately after the last word, participants were given as much time as needed to recall the words. The STM task was repeated for a total of four trials using the same randomized 12-item word list. In order to control for typing inability in some participants, all participants were asked to write their responses on a blank sheet of paper and responses were then typed into the program by the experimenter. In the RM task, which was used to measure reasoning, participants selected a missing image from an incomplete 3 × 3 matrix, based on horizontally- and vertically-progressing patterns. Participants were allotted 10 min to complete 18 problems, and performance was measured by the percent correct of total attempted problems.

### Modeling

In primary analyses, a general linear mixed-effects regression model was used to predict the TAR using age group (young vs. old), block (EO, EC, STM, and RM), total STM score, and RM percent correct. This was compared to predicting TAR without age in a hierarchical regression, using a chi-square test of nested models. Interaction affects were included for STM scores/Age/RM scores to investigate whether EEG correlates of cognitive performance may be age-dependent, and to account for the covariance between the cognitive tests (STM and RM). This model was assessed separately for each location (Fz, Cz, Pz). Participant ID was included as a random effect to account for repeated measures. To directly test the hypothesis that age modulates the relationship between cognitive performance and aging, a chi-square ANOVA test was used to compare models predicting the TAR with and without age.

In secondary analyses, descriptive statistics detailing the correlations among cognitive performance, age and EEG metrics were computed.

## Results

### Cognitive tests

#### STM

**Figure 1:**
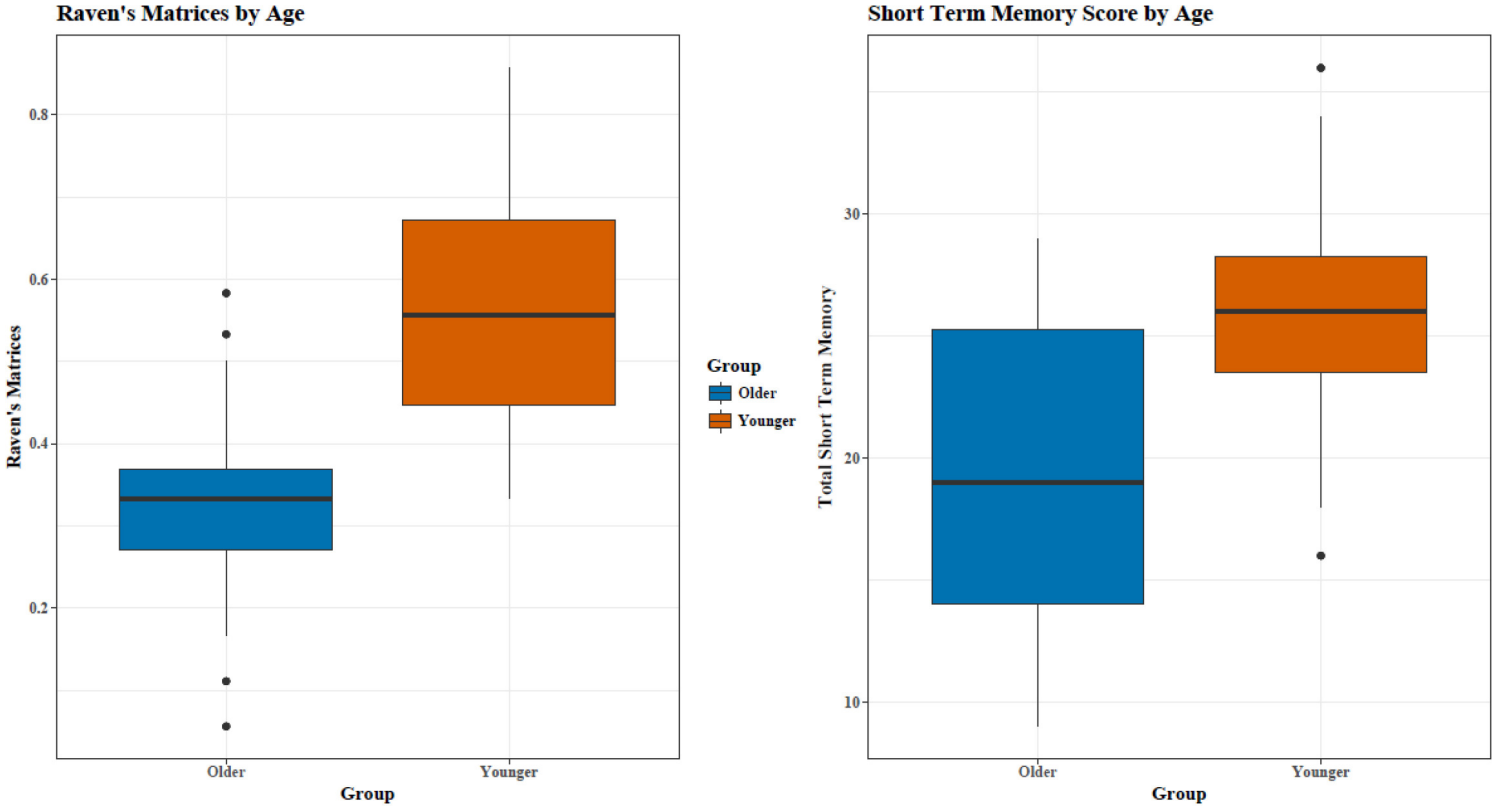
Young adults had significantly higher RM and STM scores. Outliers are represented by ^*^.

Young adults (*My* = 8.75, *SD* = 2.02) recalled more words than old adults (*Mo* = 6.70, *SD* = 2.06), *t*_(34)_ = 3.00, *p* = 0.005 across all four STM trials (see Figure 1). As seen in the *T*-tests for each trial individually, the young adults recalled significantly (*p* ≤ 0.05) or marginally (*p* ≤ 0.10) more words than old adults for each of the four trials. As results for each trial were similar, trial 4 was used for secondary correlational analyses.

- Trial 1 [*M*_*Y*_ = 4.19, *M*_*O*_ = 2.55, *t*_(34)_ = 3.82, *p* = 0.001]
- Trial 2 [*M*_*Y*_ = 5.88, *M*_*O*_ = 4.65, *t*_(34)_ = 2.41, *p* = 0.02]
- Trial 3 [*M*_*Y*_ = 7.13, *M*_*O*_ = 5.85, *t*_(34)_ = 1.72, *p* = 0.10]
- Trial 4 [*M*_*Y*_ = 8.75, *M*_*O*_ = 6.70, *t*_(34)_ = 3.00, *p* = 0.005].

#### Reasoning

**Figure 2:**
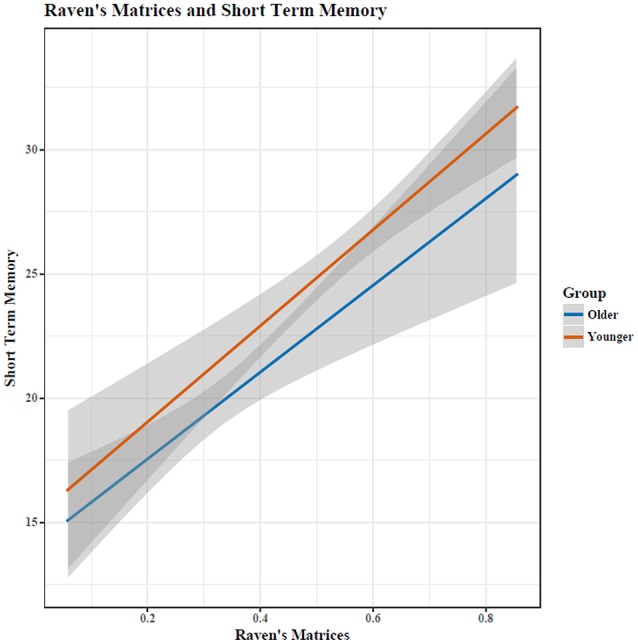
Performance on RM and STM was highly correlated (*r* = 0.6). Shaded area indicates 95% confidence intervals.

Out of 18 problems, young adults had a higher percentage of correctly completed problems [*M*_*Y*_ = 55.69, *SD* = 15.16; *M*_*O*_ = 32.57, *SD* = 12.98; *t*_(34)_ = 4.93, *p* < 0.0005, see Figure 1]. RM and STM were correlated (*r* = 0.60), with a similar relationship for both age groups (see Figure 2).

### EEG analyses

#### Primary model: TAR and cognitive performance

Cognitive performance, task, age, and gender was used to predict TAR EEG activity across blocks in three mid-line locations. For Fz (frontal) and Pz (posterior) locations, only block (EO, EC, STM, RM) was statistically significant in predicting the TAR (*p* < 0.001, Supplementary Tables 1, 2). For all regions, the EC block produced significantly lower TAR than the EO, STM, or RM blocks (*p* < 0.001).

**Figure 3:**
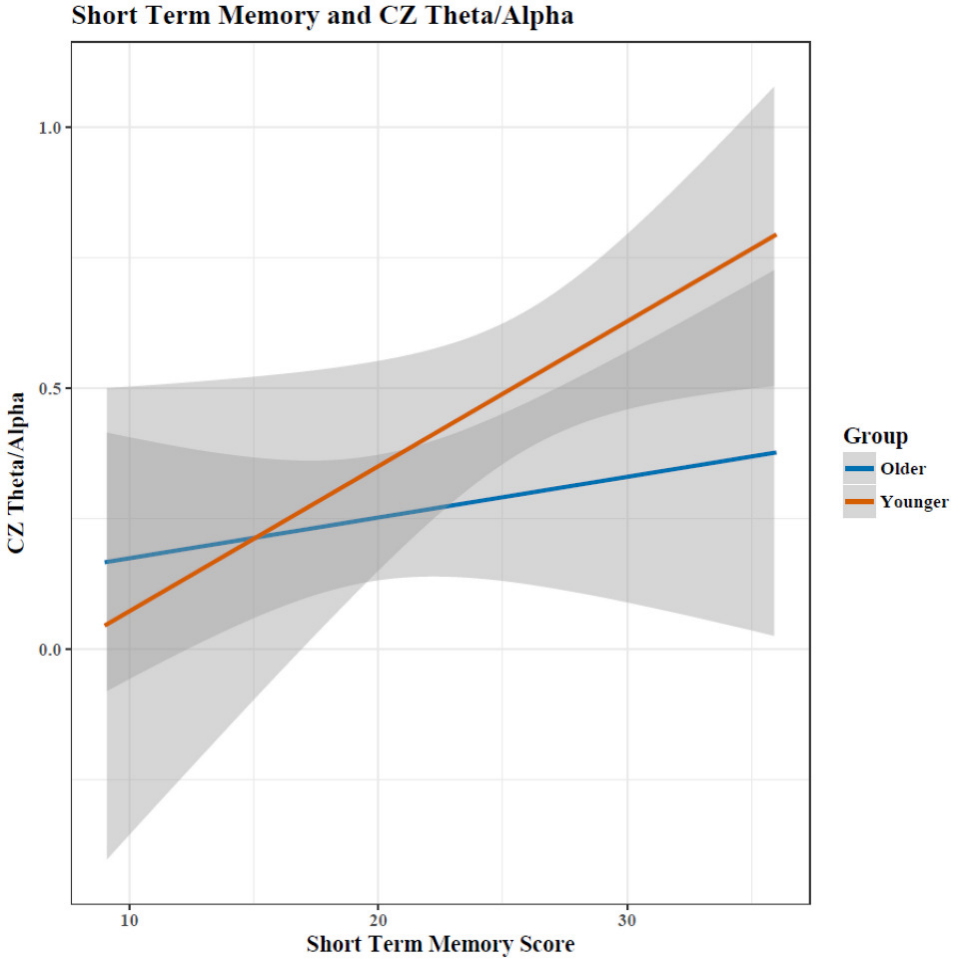
The relationship between short term memory score and the EEG Theta/Alpha ratio depended on a participant's age, with young adults showing a much larger increase in TAR with increased STM performance than old adults. Shaded area indicates 95% confidence intervals. Note: plots do not illustrate the contributing effects of other covariates (e.g., block and STM).

**Figure 4:**
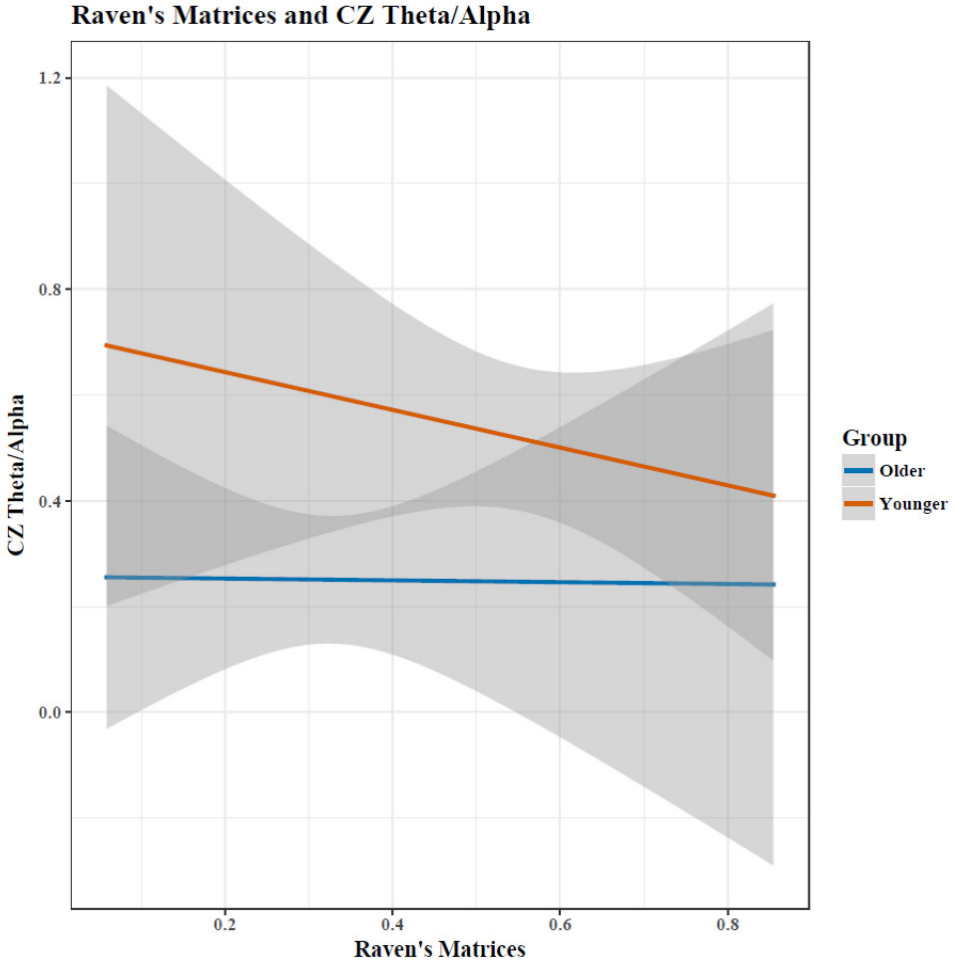
Increases in matrix reasoning score was associated with a reduced TAR (*p* < 0.05). The interaction between age and cognitive performance did not reach significance (*p* < 0.10); when cognitive performance was held constant, the TAR was greater in young than in old participants. Shaded area indicates 95% confidence intervals. Note: plots do not illustrate the contributing effects of other covariates (e.g., block and STM).

For the Cz (central) regions, the TAR was dependent upon cognitive performance only after accounting for age, determined by a hierarchical regression comparing the ability to predict the TAR with and without age (chi-square test of nested models, *p* < 0.05; Tables 1, 2). Cognitive performance significantly interacted with age in the regression model such that young adults had substantial increases in TAR with increased STM compared to old adults (*p* < 0.05; Figure 3, Table 1). For decreased RM, subjects showed increased TAR after holding constant the effects of age, STM, and task (*p* < 0.05). The interaction effect between aging and RM suggested that young adults also had increases in TAR with increasing RM compared to older adults after holding constant all else (*p* < 0.10; Table 2, Figure 4).

**Table 1:** Theta Alpha Ratio (TAR) model parameters for CZ.

**Variable**	**Estimate**	**Std. Error**	***t*-value**	**Pr(>|t|)**	**Sig**
(Intercept)	0.472	1.062	0.444	0.659	
STM Score (total)	−0.031	0.053	−0.592	0.557	
RM Score (percent correct)	−4.768	2.061	−2.314	0.026	^*^
Young Age	−2.719	3.252	−0.836	0.408	
EEG Block: EO	0.404	0.081	5.008	0.001	^***^
EEG Block: STM	0.875	0.100	8.751	0.001	^***^
EEG Block: RM	0.886	0.099	8.955	0.001	^***^
Gender: Female	0.067	0.140	0.478	0.635	
STM Score: Young Age	0.217	0.084	2.584	0.014	^*^
STM Score: RM Score	0.121	0.151	0.802	0.427	
RM Score: Young Age	8.364	4.509	1.855	0.071	.
STM Score: Young Age: RM Score	−0.363	0.184	−1.969	0.056	.

*Cognitive performance did not predict the EEG TAR measure until accounting for the effects of age (p < 0.05, chi-square ANOVA test of nested models). The TAR within an EEG block was modeled using a general linear mixed-effects regression model. Increases in STM were more strongly associated with an increased TAR (p < 0.05) in young than old adults. Increases in RM were associated with decreases in TAR. Interpretation of coefficients is with respect to a baseline of an Older Male during Eyes Closed EEG block, implying that Eyes Closed condition was significantly different than all other tasks (p < 0.001)*.

Significance codes: 0.001 “^***^”; 0.01 “^**^”; 0.05 “^*^”; 0.1 “.”

**Table 2:** Theta Alpha Ratio (TAR) model parameters for CZ without including aging effects.

**Variable**	**Estimate**	**Std. Error**	***t*-value**	**Pr(>|t|)**	**Sig**
(Intercept)	−0.605	0.292	−2.072	0.045	^*^
STM Score (total)	0.025	0.013	1.895	0.065	.
RM Score (percent correct)	−0.120	0.485	−0.248	0.805	
EEG Block: EO	0.404	0.081	5.019	0.001	^***^
EEG Block: STM	0.867	0.0987	8.788	0.001	^***^
EEG Block: RM	0.886	0.0987	8.975	0.001	^***^
Gender: Female	0.069	0.146	0.475	0.637	

*When not including the effects of age, the TAR changed with activity but did not significantly depend on cognitive performance. Interpretation of coefficients is with respect to a baseline of an Older Male during Eyes Closed EEG block, implying that Eyes Closed condition was significantly different than all other tasks (p < 0.001)*.

Significance codes: 0.001 “^***^”; 0.01 “^**^”; 0.05 “^*^”; 0.1 “.”

To assess sensitivity of these findings to the individual variation in Alpha, these models were also replicated when excluding 2 participants whose iPAF fell outside the traditional 8–12 Hz window, which did not change these findings (Supplementary Table 3). Similarly, results were consistent when using Alpha fixed at 8–12 Hz instead of separating by Alpha 1 (8–10) Hz. and Alpha 2 (10–12) Hz. windows. This replication suggests that the individual variation in Alpha did not drive the interactions between aging and cognitive performance. When modeling just Alpha separately, increased Alpha was associated with increased cognitive ability in general, but the aging effects were not statistically significant (*p* > 0.05, Supplementary Table 4).

**Figure 5:**
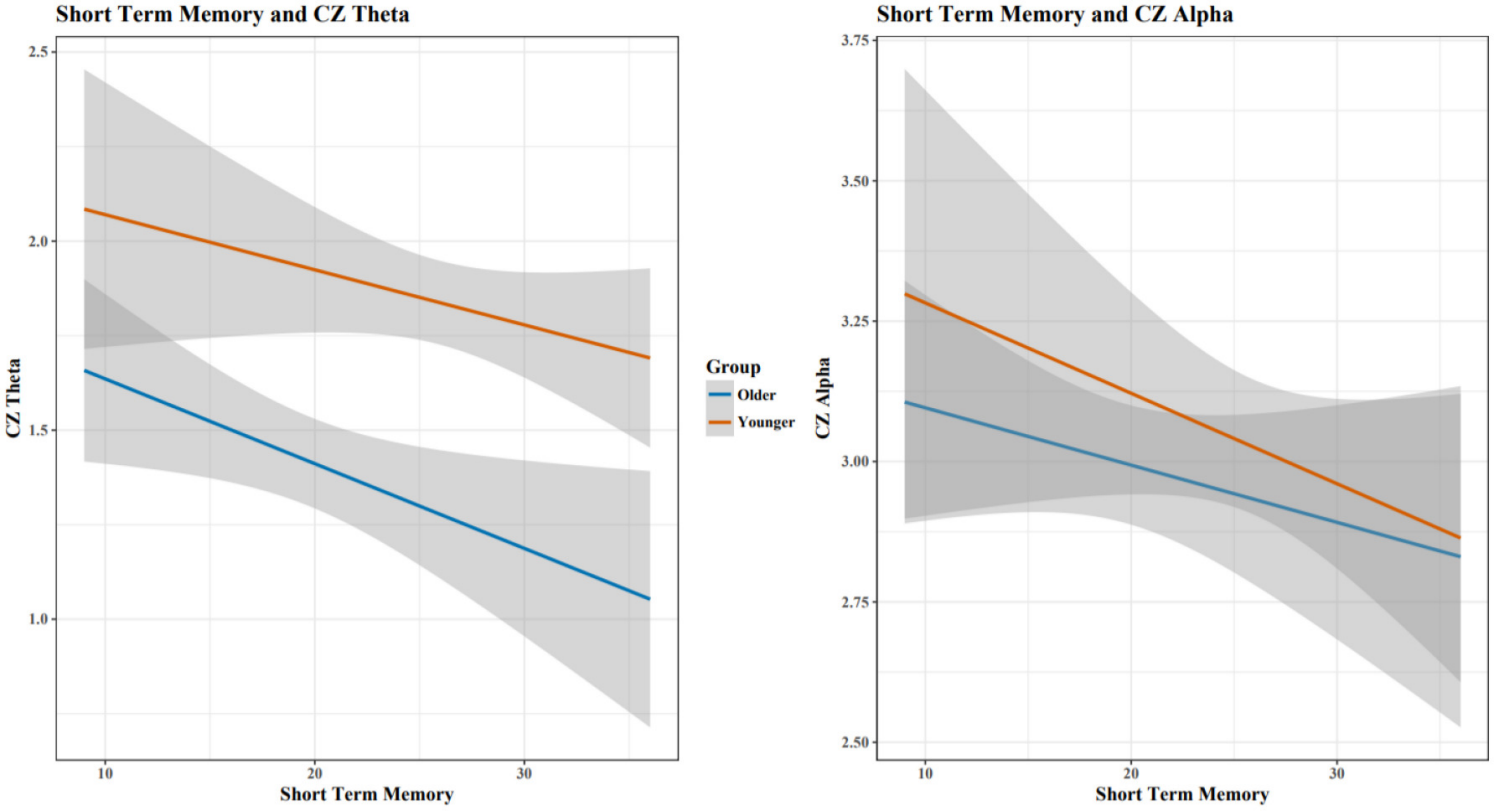
As STM scores increased, Cz Theta and Alpha decreased for both age groups. Shaded area indicates 95% confidence intervals. Plots do not illustrate the contributing effects of other covariates (e.g., block and STM).

**Figure 6:**
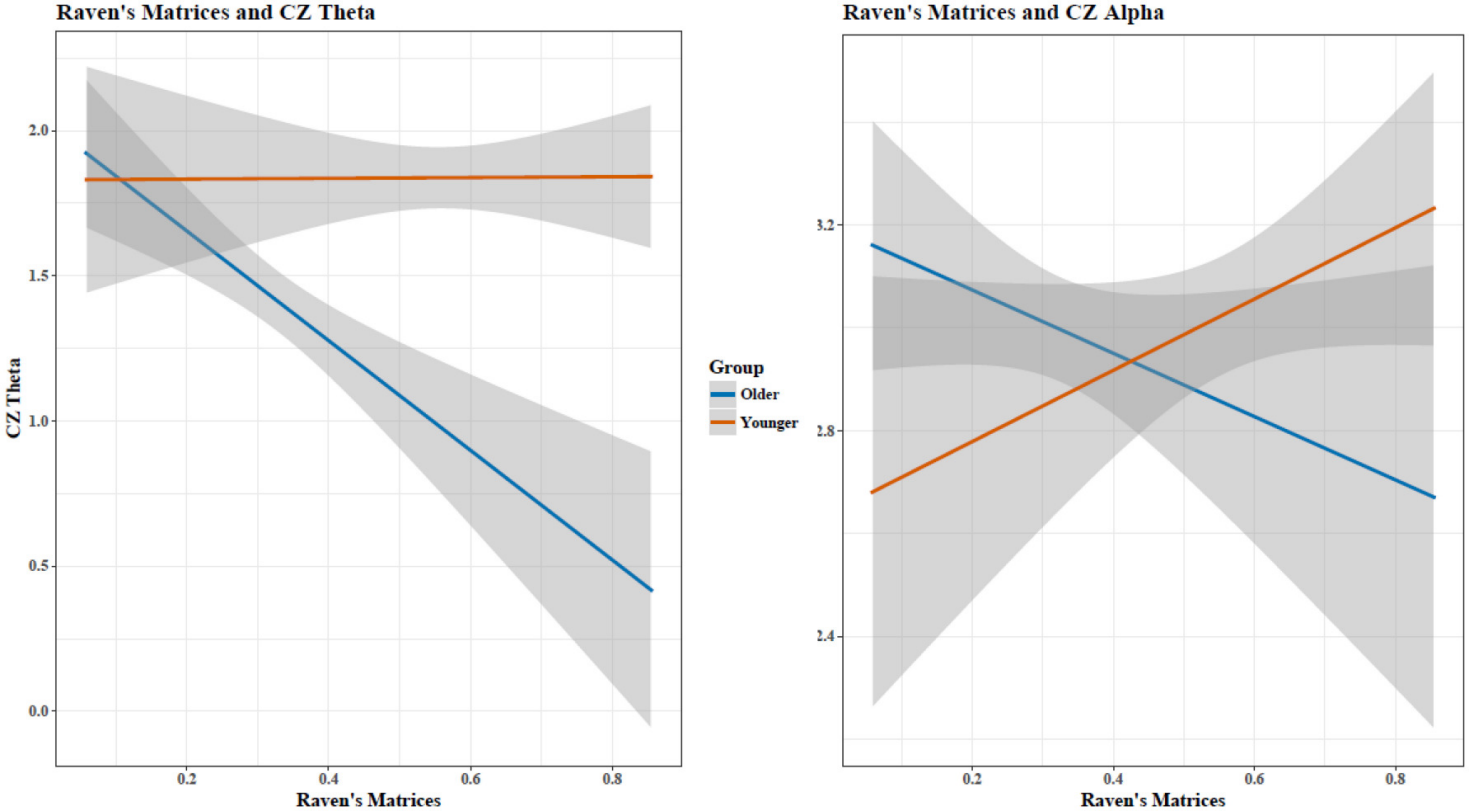
Matrix reasoning scores and Cz EEG Theta and Alpha activity showed an age-dependent relationship. Shaded area indicates 95% confidence intervals. Plots do not illustrate the contributing effects of other covariates (e.g., block and STM).

When assessing Theta and Alpha separately, increases in STM performance were associated with a decrease in Theta and Alpha for both age groups (Figure 5). Holding constant cognitive performance, young adults had greater Theta and Alpha than old adults. For RM, age showed different relationships with cognitive performance in predicting Theta and Alpha power (Figure 6), with increased RM showing increased Alpha in young adults and decreased Alpha and decreased Theta in old adults.

#### Secondary model: descriptive analyses of Fz, Cz, and Pz by age and bandwidth

EEG activity during EC1 and during cognition was correlated with iPAF, relative Delta power, relative Theta power, and relative Alpha power at the three midline sites: Fz, Cz, and Pz. The cognitive task dependent measures were trial 4 of the STM (as results for trials 1–3 were similar to trial 4) and RM percent correctly completed.

### Correlations

All correlation analyses were conducted with Pearson correlations, two-tailed tests. Statistical significance was not reached after Bonferroni correction, so these results are presented descriptively with uncorrected *p*-values; see Supplementary Tables 5–8 for correlations by age, for the entire sample, and for young and old adults separately.

#### Age

iPAF was significantly negatively correlated with age. During the EC1 condition, relative Delta was significantly negatively correlated with age at the Fz site. Relative Theta was significantly negatively correlated with age at Fz and Cz. Relative Alpha was not significantly correlated with age.

#### iPAF

iPAF was not correlated with STM performance but was significantly positively correlated with RM across the entire sample. IPAF was not correlated with performance within young or within old adults.

#### Relative delta

Across the entire sample, relative Delta was generally positively correlated with cognitive performance. During EC1, relative Delta was significantly positively correlated with STM at all three sites. During the STM task, relative Delta was significantly positively correlated with STM at Pz. RM was not correlated with relative Delta during EC1 or during the matrix task. For young adults, during EC1, relative Delta was significantly positively correlated with STM at Fz and marginally correlated at Cz and Pz. Relative Delta was not significantly correlated with performance during the STM task. Relative Delta was also not significantly correlated with RM performance during EC1 or during the RM task. For old adults, relative Delta was generally positively correlated with performance. During EC1, relative Delta was significantly positively correlated with STM at Pz and marginally positively correlated at Cz. Relative Delta during the STM task was also significantly positively correlated at Pz. Relative Delta was not correlated with RM performance.

#### Relative theta

Across the entire sample, relative Theta was positively correlated with cognitive performance, but this was sensitive to activity/block. STM performance was not significantly correlated with relative Theta during EC1 or during the STM task. During EC1, relative Theta was not significantly correlated with RM performance. During the Matrix task, relative Theta was significantly positively correlated with RM at the Fz and Cz sites. For both young and old adults separately, no correlations reached statistical significance, but relative Theta was generally negatively correlated with performance.

#### Relative alpha

Relative Alpha was generally negatively correlated with cognitive performance for the entire sample, although no correlations reached statistical significance. Correlations for young adults showed that relative Alpha was negatively correlated with performance. During EC1, relative Alpha was not significantly correlated with STM performance. During the STM task, relative Alpha was negatively correlated with STM performance at Pz. Relative Alpha was also not significantly correlated with RM performance during EC1 or during the RM task. Correlations for old adults likewise showed that relative Alpha was generally negatively correlated with performance. During EC1, relative Alpha was not correlated STM performance. During the STM task, relative Alpha was significantly negatively correlated with STM at Pz. Relative Alpha was not correlated with RM performance.

## Discussion

Consistent with the literature, young adults reliably outperformed old adults on STM and RM measures of cognitive function (Salthouse, 2012). The TAR marker of cognitive performance showed different trends for high-functioning young and old adults in the Cz region. For all locations, the most significant predictor of TAR was activity: TAR increased in EO, STM, and RM blocks compared to EC blocks (*p* < 0.001). The most likely reason for this TAR decrease during the EC block is the general increase in Alpha, occurring during wakeful relaxation with shut eyes (Barry et al., 2007).

Some differences seen between the RM and STM cognitive assessments/TAR measurement may reflect the different brain regions recruited during such cognitive tasks; matrix reasoning produces fMRI activation in right frontal and bilateral parietal regions (Prabhakaran et al., 1997), while verbal short-term memory tasks produces activation in the posterior temporal regions, supramarginal gyri, Broca's area, and dorsolateral premotor cortex (Henson et al., 2000). Different susceptibility of these brain regions to the aging process, as well as compensatory recruitment of other regions for specific cognitive tasks, also may impact the differences seen between the two assessments (STM, RM) within our old cohort. For example, patients with Alzheimer's disease showed decreased fMRI cortical activation but increased hippocampal activation during a STM task relative to controls, suggesting compensatory recruitment (Peters et al., 2009). Similarly, cortical recruitment strategies change with age; healthy elderly adults used frontal areas for a spatial working memory task, whereas healthy younger adults recruited parietal areas (McEvoy et al., 2001).

Increased TAR was associated with decreases in RM performance in old adults, with the opposite trend seen in young adults. This is consistent with the findings of Bian et al. (2014), who found that diabetic older patients with MCI had an increased TAR compared to diabetic older adults without MCI, as well as Moretti (2015) who found an increased Theta frequency power in MCI adults due to Alzheimer's disease. The increased Theta frequency was also associated with hippocampal atrophy, which suggests that a greater TAR would be associated with greater hippocampal atrophy when holding Alpha constant (Moretti, 2015).

Progressive atrophy of the hippocampus, measured through MRI, also correlated with decreased EEG cortical Alpha power in adults with Alzheimer's disease (Babiloni et al., 2009), while Penttilä et al. (1985) found that decreases in Alpha in those with Alzheimer's disease was specific to late-stage disease. Jelic et al. (1996) found cognitive impairment was associated with increased Theta and decreased Alpha power in adults with Alzheimer's disease. Although our study found also that increased Theta was associated with decreased cognitive functioning in healthy old adults, decreased Alpha was associated with increased cognitive ability for RM (Figure 6). This suggests that the relationship between cognitive ability and Alpha is non-linear, where optimal cognitive functioning occurs when Alpha shows *moderate* age-associated changes. This closely echoes earlier MEG findings of Vlahou et al. (2014), who found enhanced Delta and Theta power with increased executive functioning and perceptual speed, but only within healthy older adults, further suggesting a non-linear relationship between aging, brain activity, and cognitive functioning. Healthy aging produces changes in EEG spectra in the absence of pathology (Polich, 1997), so age-related and pathological-related power changes seen in diseases of the elderly are ultimately dependent upon the “control” group being studied.

For all participants within most blocks, cognitive performance (STM and RM) was generally positively correlated with IPAF, relative Delta, and relative Theta, and negatively correlated with relative Alpha (Supplementary Table 6), although these pairwise correlations did not surpass multiple comparison corrections and are presented descriptively. As seen in the separate correlational analyses for young adults (Supplementary Table 7), IPAF and relative Delta did not show correlations in a general direction, and both relative Theta and relative Alpha were generally negatively correlated with cognitive performance. In contrast, old adults showed general positive correlations between cognitive performance and both iPAF and relative Delta (Supplementary Table 8). Similarly, old adults showed general negative correlations between cognitive performance and both relative Theta and relative Alpha. Furthermore, correlations were generally similar both at rest (EC1) and during cognition. As relative Delta activity correlated similarly for young and old adults, our correlational data suggests that these relationships with cognitive performance remain stable regardless of age. Overall, few bivariate correlations were significant after correction for multiple comparisons, likely due to the individual blocks being underpowered because of small sample size and the use of two-tailed (non-directional) rather than one-tailed (directional) testing. Age was negatively correlated with iPAF, relative Delta, and relative Theta (Supplementary Table 5).

Overall, our correlations are consistent with other findings in regards to iPAF (Klimesch, 1997, 1999; Angelakis et al., 2004; Clark et al., 2004; Grandy et al., 2013), relative Delta (Vlahou et al., 2014), relative Theta (Jensen and Tesche, 2002; Cummins and Finnigan, 2007; Cummins et al., 2008; Vlahou et al., 2014) and findings of Alpha power inversely correlated with age (Hartikainen et al., 1992; Hong and Rebec, 2012) but inconsistent with Finnigan and Robertson (2011), who found positive relationships between relative Theta and cognitive performance in old adults, but no association of resting iPAF, relative Delta power, and relative Alpha power with cognitive performance. Thus, while our study design was a close replication of Finnigan and Robertson (2011), with the addition of a young adult group and an older age range in the old adult group (70–79 instead of 55–73), and EEG metrics recorded both at rest and during the cognitive assessment, our correlational findings align better with other studies.

Our findings have lent some clarity to the mixed results in literature regarding age, EEG, and cognitive performance, yet there are some shortcomings to our study. Because our sample size was small, this study was underpowered to detect the smaller effect-sizes in other brain regions. Thus, the insignificance of the TAR in this analyses in frontal regions (Fz) does not confirm the absence of an age-dependent cognition relationship, but may rather suggest that the effect size is smaller in Fz than that of the central region (Cz). A larger sample would also have allowed us to evaluate more hypotheses, which were purposefully few to avoid false positives due to multiple comparisons. Our findings were in a subset of high-functioning healthy adults, so EEG correlates of cognitive performance may differ in participants having an exclusionary medical diagnoses. Moreover, participants were not followed after the conclusion of the study to track incidence of exclusionary diagnoses, so some may have been in the premorbid stages during the study period. Lastly, while we deliberately chose to focus on a narrow age range for old adults (70–79), it is possible that these results are not generalizable to old adults across a larger age spectrum, which may explain the differences between our and Finnigan and Robertson (2011)'s findings which included patients as young as 55. Future research would benefit from a longitudinal analysis of adult EEG activity and cognitive performance across a larger age spectrum.

Future research should include additional measures of cognition and brain activity. For instance, the Stroop task would serve as a measure of inhibitory control, and may be associated with other EEG measures such as P3 amplitude (Saliasi et al., 2013). In addition, it is unlikely that age by itself is the only predictor of EEG activity and performance. Many other factors that vary widely in old adults, such as physical activity, education, sleep quality and quantity, and social support and interaction, are likely to co-vary with and predict cognition. Our descriptive measures of EEG (e.g., localized relative power) were purposefully selected because their simplicity makes them more clinically applicable; however, more complex global measures of EEG behavior such as coherence may yield a deeper understanding of the relationship between cognitive performance and age. This also is a direction for future work.

In conclusion, this study adds to the existing body of knowledge by illustrating that healthy aging is associated with changes in EEG activation patterns and cognitive performance. EEG markers such as the TAR may disambiguate cognitive changes specific to healthy and pathological aging. The significant interaction effects between aging and cognitive performance indicated that a failure to show age-related changes resulted in “young” EEG signatures but impaired cognitive performance. Rather than aging-related changes being a marker of detriments in cognitive performance, a “healthy” person whose EEG patterns do *not* change with age is more likely to exhibit cognitive impairment than a person who shows normal age-related changes.

## Ethics statement

This study was carried out in accordance with the recommendations of the following guidelines: APA guidelines, the Belmont Report, U.S. Department of Health and Human Services PART 46: PROTECTION OF HUMAN SUBJECTS, California Experimental Subjects Bill of Rights, and Pepperdine University's Seaver IRB, with written informed consent from all subjects. All subjects gave written informed consent in accordance with the Declaration of Helsinki. The protocol was approved by Pepperdine University's Seaver IRB.

## Author contributions

All authors meet the following authorship contribution criteria: Substantial contributions to the conception or design of the work, as well as the acquisition, analysis, and interpretation of data for the work; Drafting the work and revising it critically; Final approval of the version to be published; Agreement to be accountable for all aspects of the work in ensuring that questions related to the accuracy or integrity of any part of the work are appropriately investigated and resolved.

### Conflict of interest statement

The authors declare that the research was conducted in the absence of any commercial or financial relationships that could be construed as a potential conflict of interest.

## References

[B1] Angelakis E. Lubar J. F. Stathopoulou S. (2004). Electroencephalographic peak alpha frequency correlates of cognitive traits. Neurosci. Lett. 371, 60–63. 10.1016/j.neulet.2004.08.041 15500967

[B2] Babiloni C. Frisoni G. B. Pievani M. Vecchio F. Lizio R. Buttiglione M.. (2009). Hippocampal volume and cortical sources of EEG alpha rhythms in mild cognitive impairment and Alzheimer disease. Neuroimage 44, 123–135. 10.1016/j.neuroimage.2008.08.005 18805495

[B3] Barry R. J. Clarke A. R. Johnstone S. J. Magee C. A. Rushby J. A. (2007). EEG differences between eyes-closed and eyes-open resting conditions. Clin. Neurophysiol. 118, 2765–2773. 10.1016/j.clinph.2007.07.028 17911042

[B4] Basar E. Guntekin B. (2013). Review of delta, theta, alpha, beta, and gamma response oscillations in neuropsychiatric disorders. Suppl. Clin. Neurophysiol. 62, 303–341. 10.1016/B978-0-7020-5307-8.00019-3 24053047

[B5] Bian Z. Li Q. Wang L. Lu C. Yin S. Li X. (2014). Relative power and coherence of EEG series are related to amnestic mild cognitive impairment in diabetes. Front. Aging Neurosci. 6:11. 10.3389/fnagi.2014.00011 24550827 PMC3912457

[B6] Bousleiman H. Chaturvedi M. Gschwandtner U. Hatz F. Schindler C. Zimmermann R. (2015). P122. Alpha1/theta ratio from quantitative EEG (qEEG) as a reliable marker for mild cognitive impairment (MCI) in patients with Parkinson's disease (PD). Clin. Neurophysiol. 126, e150–e151. 10.1016/j.clinph.2015.04.249

[B7] Chiang A. K. I. Rennie C. J. Robinson P. A. Van Albada S. J. Kerr C. C. (2011). Age trends and sex differences of alpha rhythms including split alpha peaks. Clin. Neurophysiol. 122, 1505–1517. 10.1016/j.clinph.2011.01.040 21349761

[B8] Clark C. R. Veltmeyer M. D. Hamilton R. J. Simms E. Paul R. Hermens D. (2004). Spontaneous alpha peak frequency predicts working memory performance across the age span. Int. J. Psychophysiol. 53, 1–9. 10.1016/j.ijpsycho.2003.12.011 15172130

[B9] Cummins T. A. D. Broughton M. Finnigan S. (2008). Theta oscillations are affected by mild cognitive impairment and cognitive load. Int. J. Psychophysiol. 70, 75–81. 10.1016/j.ijpsycho.2008.06.002 18652854

[B10] Cummins T. D. Finnigan S. (2007). Theta power is reduced in healthy cognitive aging. Int. J. Psychophysiol. 66, 10–17. 10.1016/j.ijpsycho.2007.05.008 17582632

[B11] Finnigan S. Robertson I. H. (2011). Resting EEG theta power correlates with cognitive performance in healthy older adults. Psychophysiology 48, 1083–1087. 10.1111/j.1469-8986.2010.01173.x 21729101

[B12] Folstein M. F. (1975). “Mini-mental state”. A practical method for grading the cognitive state of patients for the clinician. J. Psychiatric Res. 12, 189–198. 10.1016/0022-3956(75)90026-6 1202204

[B13] Fonseca L. C. Tedrus G. M. Fondello M. A. Reis I. N. Fontoura D. S. (2011). EEG theta and alpha reactivity on opening the eyes in the diagnosis of Alzheimer's disease. Clin. EEG Neurosci. 42, 185–189. 10.1177/155005941104200308 21870471

[B14] Gilbert J. G. Levee R. F. (1971). Patterns of declining memory. J. Gerontol. 26, 70–75. 10.1093/geronj/26.1.70 5540322

[B15] Grandy T. H. Werkle-Bergner M. Chicherio C. Lövdén M. Schmiedek F. Lindenberger U. (2013). Individual alpha peak frequency is related to latent factors of general cognitive abilities. Neuroimage 79, 10–18. 10.1016/j.neuroimage.2013.04.059 23624490

[B16] Haegens S. Cousijn H. Wallis G. Harrison P. J. Nobre A. C. (2014). Inter-and intra-individual variability in alpha peak frequency. Neuroimage 92, 46–55. 10.1016/j.neuroimage.2014.01.049 24508648 PMC4013551

[B17] Hartikainen P. Soininen H. Partanen J. Helkala E. Riekkinen P. (1992). Aging and spectral analysis of EEG in normal subjects: a link to memory and CSF AChE. Acta Neurol. Scand. 86, 148–155. 10.1111/j.1600-0404.1992.tb05057.x 1414224

[B18] Hartman M. Bolton E. Fehnel S. E. (2001). Accounting for age differences on the Wisconsin Card Sorting Test: decreased working memory, not inflexibility. Psychol. Aging 16, 385–399. 10.1037/0882-7974.16.3.385 11554518

[B19] Henson R. N. A. Burgess N. Frith C. D. (2000). Recoding, storage, rehearsal and grouping in verbal short-term memory: an fMRI study. Neuropsychologia 38, 426–440. 10.1016/S0028-3932(99)00098-6 10683393

[B20] Hong S. L. Rebec G. V. (2012). A new perspective on behavioral inconsistency and neural noise in aging: compensatory speeding of neural communication. Front. Aging Neurosci. 4:27. 10.3389/fnagi.2012.00027 23055970 PMC3457006

[B21] Hurd M. D. Martorell P. Delavande A. Mullen K. J. Langa K. M. (2013). Monetary costs of dementia in the United States. N. Engl. J. Med. 368, 1326–1334. 10.1056/NEJMsa1204629 23550670 PMC3959992

[B22] Jelic V. Johansson S. E. Almkvist O. Shigeta M. Julin P. Nordberg A.. (2000). Quantitative electroencephalography in mild cognitive impairment: longitudinal changes and possible prediction of Alzheimer's disease. Neurobiol. Aging 21, 533–540. 10.1016/S0197-4580(00)00153-6 10924766

[B23] Jelic V. Shigeta M. Julin P. Almkvist O. Winblad B. Wahlund L. O. (1996). Quantitative electroencephalography power and coherence in Alzheimer's disease and MCI. Dementia 7, 314–323. 8915037 10.1159/000106897

[B24] Jensen O. Tesche C. D. (2002). Frontal theta activity in humans increases with memory load in a working memory task. Eur. J. Neurosci. 15, 1395–1399. 10.1046/j.1460-9568.2002.01975.x 11994134

[B25] Klimesch W. (1997). EEG-alpha rhythms and memory processes. Int. J. Psychophysiol. 26, 319–340. 10.1016/S0167-8760(97)00773-3 9203012

[B26] Klimesch W. (1999). EEG alpha and theta oscillations reflect cognitive and memory performance: a review and analysis. Brain Res. Rev. 29, 169–195. 10.1016/S0165-0173(98)00056-3 10209231

[B27] Lithfous S. Tromp D. Dufour A. Pebayle T. Goutagny R. Després O. (2015). Decreased theta power at encoding and cognitive mapping deficits in elderly individuals during a spatial memory task. Neurobiol. Aging 36, 2821–2829 10.1016/j.neurobiolaging.2015.07.007 26248864

[B28] McEvoy L. K. Pellouchoud E. Smith M. E. Gevins A. (2001). Neurophysiological signals of working memory in normal aging. Cogn. Brain Res. 11, 363–376. 10.1016/S0926-6410(01)00009-X 11339986

[B29] Montez T. Poil S. S. Jones B. F. Manshanden I. Verbunt J. P. van Dijk B. W.. (2009). Altered temporal correlations in parietal alpha and prefrontal theta oscillations in early-stage Alzheimer disease. Proc. Natl. Acad. Sci. U.S.A. 106, 1614–1619. 10.1073/pnas.0811699106 19164579 PMC2635782

[B30] Moretti D. V. (2015). Theta and alpha EEG frequency interplay in subjects with mild cognitive impairment: evidence from EEG, MRI, and SPECT brain modifications. Front. Aging Neurosci. 7:31. 10.3389/fnagi.2015.00031 25926789 PMC4396516

[B31] Parkin A. J. Walter B. M. (1991). Aging, short-term memory, and frontal dysfunction. Psychobiology 19, 175–179.

[B32] Penttilä M. Partanen J. V. Soininen H. Riekkinen P. J. (1985). Quantitative analysis of occipital EEG in different stages of Alzheimer's disease. Electroencephalogr. Clin. Neurophysiol. 60, 1–6. 10.1016/0013-4694(85)90942-3 2578347

[B33] Peters F. Collette F. Degueldre C. Sterpenich V. Majerus S. Salmon E. (2009). The neural correlates of verbal short-term memory in Alzheimer's disease: an fMRI study. Brain 132, 1833–1846. 10.1093/brain/awp075 19433442

[B34] Plassman B. Langa K. Steffens D. Willis R. Wallace R. Fisher G.. (2007). Prevalence of dementia in the United States: the aging, demographics, and memory study. Neuroepidemiology 29, 125–132. 10.1159/000109998 17975326 PMC2705925

[B35] Polich J. (1997). EEG and ERP assessment of normal aging. Electroencephalogr. Clin. Neurophysiol. Evok. Potentials Sect. 104, 244–256. 10.1016/S0168-5597(97)96139-6 9186239

[B36] Prabhakaran V. Smith J. A. Desmond J. E. Glover G. H. Gabrieli J. D. (1997). Neural substrates of fluid reasoning: an fMRI study of neocortical activation during performance of the Raven's Progressive Matrices Test. Cogn. Psychol. 33, 43–63. 9212721 10.1006/cogp.1997.0659

[B37] Prichep L. S. John E. R. Ferris S. H. Rausch L. Fang Z. Cancro R.. (2006). Prediction of longitudinal cognitive decline in normal elderly with subjective complaints using electrophysiological imaging. Neurobiol. Aging 27, 471–481. 10.1016/j.neurobiolaging.2005.07.021 16213630

[B38] Salat D. H. Kaye J. A. Janowsky J. S. (2002). Greater orbital prefrontal volume selectively predicts worse working memory performance in older adults. Cereb. Cortex 12, 494–505. 10.1093/cercor/12.5.494 11950767

[B39] Saliasi E. Geerligs L. Lorist M. M. Maurits N. M. Rypma B. (2013). The relationship between P3 amplitude and working memory performance differs in young and older Adults. PLoS ONE 8:e63701. 10.1371/journal.pone.0063701 23667658 PMC3646823

[B40] Salthouse T. (2012). Consequences of age-related cognitive declines. Annu. Rev. Psychol. 63, 201–226. 10.1146/annurev-psych-120710-100328 21740223 PMC3632788

[B41] Salthouse T. A. (1990). Working memory as a processing resource in cognitive aging. Develop. Rev. 10, 101–124. 10.1016/0273-2297(90)90006-P

[B42] Schmidt M. Kanda P. Basile L. da Silva Lopes H. F. Baratho R. Demario J.. (2013). Index of alpha/theta ratio of the electroencephalogram: a new marker for Alzheimer's disease. Front. Aging Neurosci. 5:60. 10.3389/fnagi.2013.00060 24130529 PMC3793211

[B43] Sheikh J. I. Yesavage J. A. (1986). Geriatric depression scale (GDS): recent evidence and development of a shorter version. Clin. Gerontol. 5, 165–173. 10.1300/J018v05n01_09

[B44] Streletz L. J. Reyes P. F. Zalewska M. Katz L. Fariello R. G. (1990). Computer analysis of EEG activity in dementia of the Alzheimer's type and Huntington's disease. Neurobiol. Aging 11, 15–20. 10.1016/0197-4580(90)90057-7 2139184

[B45] US Census Bureau (2016). Facts for Features: Older Americans Month: May 2016 (Release Number: CB16-FF.08). Available online at: http://www.census.gov/newsroom/facts-for-features/2016/cb16-ff08.html

[B46] Vlahou E. L. Thurm F. Kolassa I. T. Schlee W. (2014). Resting-state slow wave power, healthy aging and cognitive performance. Sci. Rep. 4:5101. 10.1038/srep05101 24869503 PMC4037748

[B47] Yokota M. Miyanaga K. Yonemura K. Watanabe H. Nagashima K. Naito K.. (2000). Declining of memory functions of normal elderly persons. Psychiatry Clin. Neurosci. 54, 217–225. 10.1046/j.1440-1819.2000.00662.x 10803819

