# Supplementary material for: The Relationship of Cognitive Performance and the Theta-Alpha Power Ratio Is Age-Dependent: An EEG Study of Short Term Memory and Reasoning during Task and Resting-State in Healthy Young and Old Adults
Source: Front Aging Neurosci. 2017 Nov 7;9:364. doi: 10.3389/fnagi.2017.00364 (PMC5682032; doi:10.3389/fnagi.2017.00364)
Supplement: Supplementary file 1 [file DataSheet1.docx]

Supplementary Table 1.

*TAR model parameters for FZ.*

| **Variable** | **Estimate** | **Std. Error** | **t value** | **Pr(>\|t\|)** | **Sig** |
| --- | --- | --- | --- | --- | --- |
| (Intercept) | 0.828 | 1.025 | 0.808 | 0.419 |  |
| STM Score (total) | -0.049 | 0.051 | -0.959 | 0.338 |  |
| RM Score (percent correct) | -2.714 | 1.988 | -1.365 | 0.173 |  |
| Young Age | -3.063 | 3.137 | -0.976 | 0.329 |  |
| EEG Block: EO | 0.393 | 0.074 | 5.274 | 0.001 | *** |
| EEG Block: STM | 0.854 | 0.092 | 9.265 | 0.001 | *** |
| EEG Block: RM | 0.872 | 0.091 | 9.568 | 0.001 | *** |
| Gender: Female | 0.070 | 0.135 | 0.514 | 0.607 |  |
| STM Score: Young Age | 0.142 | 0.081 | 1.756 | 0.079 | . |
| STM Score: RM Score | 0.144 | 0.145 | 0.993 | 0.321 |  |
| RM Score: Young Age | 4.646 | 4.351 | 1.068 | 0.286 |  |
| STM Score: Young Age: RM Score | -0.227 | 0.178 | -1.275 | 0.203 |  |

*Note*. The Theta Alpha Ratio (TAR) was modeled using a general linear mixed-effects regression model, across all blocks. For the FZ region in young adults the Theta/Alpha ratio was increased for greater cognitive functioning (p<0.001) whereas in old adults the Theta/Alpha power ratio was decreased for greater cognitive ability.

Significance codes: ‘***’ 0.001 ‘**’ 0.01 ‘*’ 0.05 ‘.’ 0.1 ‘ ’ 1

Supplementary Table 2.

*TAR model parameters for PZ.*

| **Variable** | **Estimate** | **Std. Error** | **t value** | **Pr(>\|t\|)** | **Sig** |
| --- | --- | --- | --- | --- | --- |
| (Intercept) | -1.053 | 1.429 | -0.737 | 0.465 |  |
| STM Score (total) | 0.034 | 0.071 | 0.470 | 0.641 |  |
| RM Score (percent correct) | -4.687 | 2.772 | -1.691 | 0.099 | . |
| Young Age | 0.635 | 4.374 | 0.145 | 0.885 |  |
| EEG Block: EO | 0.415 | 0.096 | 4.330 | 0.001 | *** |
| EEG Block: STM | 1.143 | 0.119 | 9.636 | 0.001 | *** |
| EEG Block: RM | 1.130 | 0.117 | 9.635 | 0.001 | *** |
| Gender: Female | -0.017 | 0.189 | -0.091 | 0.928 |  |
| STM Score: Young Age | 0.199 | 0.113 | 1.765 | 0.085 | . |
| STM Score: RM Score | -0.035 | 0.202 | -0.172 | 0.864 |  |
| RM Score: Young Age | 6.406 | 6.066 | 1.056 | 0.297 |  |
| STM Score: Young Age: RM Score | -0.288 | 0.248 | -1.161 | 0.253 |  |

*Note*. The Theta Alpha Ratio (TAR) was modeled using a general linear mixed-effects regression model, across all blocks. For the PZ region, the TAR depended largely on activity (p<0.001). Interpretation of coefficients is with respect to a baseline of an older male subject during Eyes Closed block.

Significance codes: ‘***’ 0.001 ‘**’ 0.01 ‘*’ 0.05 ‘.’ 0.1 ‘ ’ 1

Supplementary Table 3.

*TAR model parameters for CZ with two participants removed.*

| **Variable** | **Estimate** | **Std. Error** | **t value** | **Pr(>\|t\|)** | **Sig** |
| --- | --- | --- | --- | --- | --- |
| (Intercept) | 0.431 | 1.109 | 0.389 | 0.699 |  |
| STM Score (total) | -0.029 | 0.055 | -0.526 | 0.602 |  |
| RM Score (percent correct) | -4.727 | 2.140 | -2.209 | 0.033 | * |
| Young Age | -2.582 | 3.441 | -0.750 | 0.458 |  |
| EEG Block: EO | 0.405 | 0.085 | 4.785 | 0.001 | *** |
| EEG Block: RM | 0.876 | 0.104 | 8.461 | 0.001 | *** |
| EEG Block: STM | 0.881 | 0.104 | 8.511 | 0.001 | *** |
| Gender: Female | 0.068 | 0.153 | 0.445 | 0.659 |  |
| STM Score: Young Age | 0.214 | 0.087 | 2.460 | 0.018 | * |
| STM Score: RM Score | 0.115 | 0.159 | 0.724 | 0.473 |  |
| RM Score: Young Age | 8.225 | 4.719 | 1.743 | 0.089 | . |
| STM Score: Young Age: RM Score | -0.357 | 0.193 | -1.851 | 0.072 | . |

*Note*: Removing two participants whose iPAF fell outside the traditional 8-12 HZ did not change significance of the model. Interpretation of coefficients is with respect to a baseline of an older male subject during Eyes Closed block.

Significance codes: 0.001 ‘***’; 0.01 ‘**’; 0.05 ‘*’; 0.1 ‘. ’

Supplementary Table 4.

*Alpha model parameters for CZ.*

| **Variable** | **Estimate** | **Std. Error** | **t value** | **Pr(>\|t\|)** | **Sig** |
| --- | --- | --- | --- | --- | --- |
| (Intercept) | 2.308 | 0.973 | 2.373 | 0.023 | * |
| STM Score (total) | 0.058 | 0.049 | 1.195 | 0.239 |  |
| RM Score (percent correct) | 3.510 | 2.978 | 1.179 | 0.245 |  |
| Young Age | 2.740 | 1.888 | 1.451 | 0.155 |  |
| EEG Block: EO | -0.277 | 0.073 | -3.793 | 0.001 | *** |
| EEG Block: RM | -0.702 | 0.089 | -7.848 | 0.001 | *** |
| EEG Block: STM | -0.574 | 0.089 | -6.415 | 0.001 | *** |
| Gender: Female | -0.023 | 0.128 | -0.182 | 0.857 |  |
| STM Score: Young Age | -0.150 | 0.077 | -1.956 | 0.057 | . |
| STM Score: RM Score | -0.190 | 0.138 | -1.381 | 0.175 |  |
| RM Score: Young Age | -4.858 | 4.131 | -1.176 | 0.247 |  |
| STM Score: Young Age: RM Score | 0.287 | 0.169 | 1.701 | 0.097 | . |

*Note*: Younger subjects overall had a greater Alpha than older subjects. Cognitive ability was positively associated with increased Alpha, but these effects did not surpass statistical significance. Interpretation of coefficients is with respect to a baseline of an older male subject during Eyes Closed block, demonstrating a substantial increase in Alpha during Eyes Closed compared to all other conditions (p<0.001).

Significance codes: 0.001 ‘***’; 0.01 ‘**’; 0.05 ‘*’; 0.1 ‘. ’

Supplementary Table 5.

*Correlations of EEG Activity with Age for all Participants during the Eyes Closed Condition (EC1).*

| **EEG Marker** | **Site** | ***R*** | ***p*** | | | **Sig (two-tailed)** | |
| --- | --- | --- | --- | --- | --- | --- | --- |
| iPAF |  | -.337 | | .045 | * | |  |
| Relative Delta | Fz | -.334 | | .046 | * | |  |
| Relative Delta | Cz | -.267 | | .115 |  | |  |
| Relative Delta | Pz | -.053 | | .761 |  | |  |
| Relative Theta | Fz | -.376 | | .024 | * | |  |
| Relative Theta | Cz | -.410 | | .013 | ** | |  |
| Relative Theta | Pz | -.160 | | .351 |  | |  |
| Relative Alpha | Fz | .077 | | .657 |  | |  |
| Relative Alpha | Cz | .036 | | .834 |  | |  |
| Relative Alpha | Pz | -.052 | | .765 |  | |  |

*Note*. Individual peak Alpha frequency (iPAF) was calculated by evaluating the maximal difference peak (6.0-13.5 Hz) in occipital and parietal electrode sites during Alpha suppression (EC1-EO1).

Significance codes: 0.001 ‘***’; 0.01 ‘**’; 0.05 ‘*’; 0.1 ‘. ’

Supplementary Table 6.

*Correlations of EEG Activity with Cognitive Performance for all Participants.*

| **EEG Marker** | **Site** | **Block** | **Task** | ***R*** | ***p*** | **Sig (two-tailed)** | |
| --- | --- | --- | --- | --- | --- | --- | --- |
| iPAF |  |  | STM | .222 | .192 | |  |
| iPAF |  |  | RM | .346 | .039 | | * |
| Relative Delta | Fz | EC1 | STM | .425 | .010 | | ** |
| Relative Delta | Fz | EC1 | RM | .164 | .340 | |  |
| Relative Delta | Fz | STM | STM | .015 | .931 | |  |
| Relative Delta | Fz | RM | RM | -.104 | .545 | |  |
| Relative Delta | Cz | EC1 | STM | .480 | .003 | | ** |
| Relative Delta | Cz | EC1 | RM | .086 | .620 | |  |
| Relative Delta | Cz | STM | STM | .160 | .359 | |  |
| Relative Delta | Cz | RM | RM | .071 | .680 | |  |
| Relative Delta | Pz | EC1 | STM | .438 | .008 | | ** |
| Relative Delta | Pz | EC1 | RM | -.021 | .903 | |  |
| Relative Delta | Pz | STM | STM | .363 | .032 | | * |
| Relative Delta | Pz | RM | RM | .055 | .750 | |  |
| Relative Theta | Fz | EC1 | STM | .028 | .873 | |  |
| Relative Theta | Fz | EC1 | RM | .270 | .112 | |  |
| Relative Theta | Fz | STM | STM | .121 | .488 | |  |
| Relative Theta | Fz | RM | RM | .465 | .004 | | ** |
| Relative Theta | Cz | EC1 | STM | .066 | .701 | |  |
| Relative Theta | Cz | EC1 | RM | .257 | .131 | |  |
| Relative Theta | Cz | STM | STM | .212 | .221 | |  |
| Relative Theta | Cz | RM | RM | .356 | .033 | | * |
| Relative Theta | Pz | EC1 | STM | .016 | .924 | |  |
| Relative Theta | Pz | EC1 | RM | .082 | .636 | |  |
| Relative Theta | Pz | STM | STM | .081 | .642 | |  |
| Relative Theta | Pz | RM | RM | .244 | .152 | |  |
| Relative Alpha | Fz | EC1 | STM | -.269 | .113 | |  |
| Relative Alpha | Fz | EC1 | RM | .022 | .901 | |  |
| Relative Alpha | Fz | STM | STM | -.128 | .465 | |  |
| Relative Alpha | Fz | RM | RM | -.065 | .707 | |  |
| Relative Alpha | Cz | EC1 | STM | -.279 | .100 | | . |
| Relative Alpha | Cz | EC1 | RM | .074 | .667 | |  |
| Relative Alpha | Cz | STM | STM | -.264 | .125 | |  |
| Relative Alpha | Cz | RM | RM | -.103 | .552 | |  |
| Relative Alpha | Pz | EC1 | STM | -.261 | .124 | |  |
| Relative Alpha | Pz | EC1 | RM | .114 | .509 | |  |
| Relative Alpha | Pz | STM | STM | -.314 | .066 | | . |
| Relative Alpha | Pz | RM | RM | -.024 | .891 | |  |

Supplementary Table 7.

*Statistics for Correlations of EEG Activity with Cognitive Performance for Young Adults.*

| **EEG Marker** | **Site** | **Block** | **Task** | ***R*** | ***p*** | **Sig (two-tailed)** | |
| --- | --- | --- | --- | --- | --- | --- | --- |
| iPAF |  |  | STM | .183 | .498 | |  |
| iPAF |  |  | RM | -.085 | .754 | |  |
| Relative Delta | Fz | EC1 | STM | .492 | .053 | | * |
| Relative Delta | Fz | EC1 | RM | -.289 | .278 | |  |
| Relative Delta | Fz | STM | STM | -.408 | .116 | |  |
| Relative Delta | Fz | RM | RM | .151 | .576 | |  |
| Relative Delta | Cz | EC1 | STM | .421 | .104 | | . |
| Relative Delta | Cz | EC1 | RM | -.389 | .137 | |  |
| Relative Delta | Cz | STM | STM | -.334 | .206 | |  |
| Relative Delta | Cz | RM | RM | .114 | .673 | |  |
| Relative Delta | Pz | EC1 | STM | .454 | .077 | | . |
| Relative Delta | Pz | EC1 | RM | -.322 | .225 | |  |
| Relative Delta | Pz | STM | STM | .081 | .765 | |  |
| Relative Delta | Pz | RM | RM | .030 | .913 | |  |
| Relative Theta | Fz | EC1 | STM | .283 | .226 | |  |
| Relative Theta | Fz | EC1 | RM | -.113 | .636 | |  |
| Relative Theta | Fz | STM | STM | -.328 | .170 | |  |
| Relative Theta | Fz | RM | RM | .077 | .747 | |  |
| Relative Theta | Cz | EC1 | STM | -.239 | .310 | |  |
| Relative Theta | Cz | EC1 | RM | -.137 | .565 | |  |
| Relative Theta | Cz | STM | STM | -.235 | .333 | |  |
| Relative Theta | Cz | RM | RM | -.027 | .912 | |  |
| Relative Theta | Pz | EC1 | STM | -.054 | .822 | |  |
| Relative Theta | Pz | EC1 | RM | -.067 | .779 | |  |
| Relative Theta | Pz | STM | STM | -.140 | .566 | |  |
| Relative Theta | Pz | RM | RM | -.167 | .482 | |  |
| Relative Alpha | Fz | EC1 | STM | -.129 | .588 | |  |
| Relative Alpha | Fz | EC1 | RM | -.175 | .460 | |  |
| Relative Alpha | Fz | STM | STM | -.263 | .276 | |  |
| Relative Alpha | Fz | RM | RM | -.106 | .656 | |  |
| Relative Alpha | Cz | EC1 | STM | -.213 | .367 | |  |
| Relative Alpha | Cz | EC1 | RM | -.158 | .507 | |  |
| Relative Alpha | Cz | STM | STM | -.329 | .169 | |  |
| Relative Alpha | Cz | RM | RM | -.231 | .327 | |  |
| Relative Alpha | Pz | EC1 | STM | -.302 | .195 | |  |
| Relative Alpha | Pz | EC1 | RM | -.187 | .429 | |  |
| Relative Alpha | Pz | STM | STM | -.520 | .023 | | * |
| Relative Alpha | Pz | RM | RM | -.194 | .413 | |  |

Supplementary Table 8.

*Statistics for Correlations of EEG Activity with Cognitive Performance for Old Adults.*

| **EEG Marker** | **Site** | **Block** | **Task** | ***R*** | ***p*** | **Sig (two-tailed)** | |
| --- | --- | --- | --- | --- | --- | --- | --- |
| iPAF |  |  | STM | .025 | .916 | |  |
| iPAF |  |  | RM | .346 | .135 | |  |
| Relative Delta | Fz | EC1 | STM | .229 | .332 | |  |
| Relative Delta | Fz | EC1 | RM | .069 | .772 | |  |
| Relative Delta | Fz | STM | STM | .280 | .246 | |  |
| Relative Delta | Fz | RM | RM | -.030 | .901 | |  |
| Relative Delta | Cz | EC1 | STM | .414 | .070 | | . |
| Relative Delta | Cz | EC1 | RM | .094 | .694 | |  |
| Relative Delta | Cz | STM | STM | .351 | .141 | |  |
| Relative Delta | Cz | RM | RM | .061 | .798 | |  |
| Relative Delta | Pz | EC1 | STM | .468 | .037 | | * |
| Relative Delta | Pz | EC1 | RM | .152 | .521 | |  |
| Relative Delta | Pz | STM | STM | .486 | .035 | | * |
| Relative Delta | Pz | RM | RM | .093 | .698 | |  |
| Relative Theta | Fz | EC1 | STM | -.283 | .226 | |  |
| Relative Theta | Fz | EC1 | RM | -.113 | .636 | |  |
| Relative Theta | Fz | STM | STM | -.328 | .170 | |  |
| Relative Theta | Fz | RM | RM | .077 | .747 | |  |
| Relative Theta | Cz | EC1 | STM | -.239 | .310 | |  |
| Relative Theta | Cz | EC1 | RM | -.137 | .565 | |  |
| Relative Theta | Cz | STM | STM | -.235 | .333 | |  |
| Relative Theta | Cz | RM | RM | -.027 | .912 | |  |
| Relative Theta | Pz | EC1 | STM | -.054 | .822 | |  |
| Relative Theta | Pz | EC1 | RM | -.067 | .779 | |  |
| Relative Theta | Pz | STM | STM | -.140 | .566 | |  |
| Relative Theta | Pz | RM | RM | -.167 | .482 | |  |
| Relative Alpha | Fz | EC1 | STM | -.129 | .588 | |  |
| Relative Alpha | Fz | EC1 | RM | -.175 | .460 | |  |
| Relative Alpha | Fz | STM | STM | -.263 | .276 | |  |
| Relative Alpha | Fz | RM | RM | -.106 | .656 | |  |
| Relative Alpha | Cz | EC1 | STM | -.213 | .367 | |  |
| Relative Alpha | Cz | EC1 | RM | -.158 | .447 | |  |
| Relative Alpha | Cz | STM | STM | -.329 | .167 | |  |
| Relative Alpha | Cz | RM | RM | -.231 | .327 | |  |
| Relative Alpha | Pz | EC1 | STM | -.302 | .195 | |  |
| Relative Alpha | Pz | EC1 | RM | -.187 | .429 | |  |
| Relative Alpha | Pz | STM | STM | -.520 | .023 | | * |
| Relative Alpha | Pz | RM | RM | -.194 | .413 | |  |

Appendix A

Words Used in the Short Term Memory Task:

Check

Back

Knob

Polar

Hallow

Flash

Strap

Mountain

Scrape

Brace

Engine

Origin
